# Sensillar expression and responses of olfactory receptors reveal different peripheral coding in two *Helicoverpa* species using the same pheromone components

**DOI:** 10.1038/srep18742

**Published:** 2016-01-08

**Authors:** Hetan Chang, Mengbo Guo, Bing Wang, Yang Liu, Shuanglin Dong, Guirong Wang

**Affiliations:** 1 State Key Laboratory for Biology of Plant Diseases and Insect Pests, Institute of Plant Protection, Chinese Academy of Agricultural Sciences, Beijing, 100193, China; 2 College of Plant Protection, Nanjing Agricultural University/Key Laboratory of Integrated Management of Crop Diseases and Pests (Nanjing Agricultural University), Ministry of Education, Nanjing, 210095, China

## Abstract

Male moths efficiently recognize conspecific sex pheromones thanks to their highly accurate and specific olfactory system. The *Heliothis*/*Helicoverpa* species are regarded as good models for studying the perception of sex pheromones. In this study, we performed a series of experiments to investigate the peripheral mechanisms of pheromone coding in two-closely related species, *Helicoverpa armigera* and *H. assulta*. The morphology and distribution patterns of sensilla trichoidea are similar between the two species when observed at the scanning electron microscope, but their performances are different. In *H. armigera*, three functional types of sensilla trichoidea (A, B and C) were found to respond to different pheromone components, while in *H. assulta* only two types of such sensilla (A and C) could be detected. The response profiles of all types of sensilla trichoidea in the two species well matched the specificities of the pheromone receptors (PRs) expressed in the same sensilla, as measured in voltage-clamp experiments. The expressions of PRs in neighboring olfactory sensory neurons (OSNs) within the same trichoid sensillum were further confirmed by *in situ* hybridization. Our results show how the same pheromone components can code for different messages at the periphery of two *Helicoverpa* species.

Within insect olfaction, the detection of specific pheromones is a particularly attractive system to investigate. In fact, sensitivity to pheromones is much higher that to environmental odors, making the electrophysiological responses to these stimuli much easier to record. In Lepidoptera, the signals in response to sex pheromones are detected through specifically tuned receptors and processed in dedicated areas of the antennal lobes (AL)^1^,^2^,^3^,^4^. In Lepidoptera, sex pheromone components are usually detected in sensilla trichodea, which represent the majority of sensory hairs on the male antenna and house the dendrites of 2–3 olfactory sensory neurons (OSNs)^5^,^6^. Several studies have provided evidence that moth peripheral integration of pheromone components information can occur in sensilla trichodea^7^,^8^,^9^.

Among the different proteins involved in insect olfaction, odorant binding proteins (OBPs), chemosensory proteins (CSPs), odorant receptors (ORs), ionotropic receptors (IRs), and the sensory neuron membrane proteins (SNMPs)^3^,^10^,^11^, ORs play a central role in the activation of OSNs. ORs are trans-membrane proteins located in the dendrite membrane of OSNs showing a “reversed” topology with their N-terminus inside the cell and the C-terminus exposed to the external environment compared with mammal ORs. Individual ORs work in tandem with a common member, named Orco (OR co-receptor), likely by forming heterodimeric structures.

*Helicoverpa armigera* and *Helicoverpa assulta* are two closely related species and represent serious pests in China and other countries. *H. armigera* is polyphagous and feeds on more than 60 crops, such as cotton, corn, wheat, soybean, tomato and other solanaceous species, while *H. assulta* is an oligophagous species mainly feeding on tobacco^12^. The two species are morphologically similar and share the same sex pheromone components^13^. Interspecific hybridization had been accomplished in the lab, producing in some cases viable offspring^14^,^15^. The two main components of their sex pheromones are (*Z*)-11-hexadecenal (Z11-16:Ald) and (*Z*)-9-hexadecenal (Z9-16:Ald), used by the two species in nearly reversed ratios, 100:2 by *H. armigera* and 6:100 by *H. assulta*, thus ensuring segregation in nature^16^,^17^.

In addition, the females of both species also produce other components in their pheromone glands including (*Z*)-9-tetradecanal (Z9-14:Ald), hexadecane-1-ol (16:OH), (*Z*)-9-hexadecanol (Z9-16:OH), (*Z*)-11-hexadecanol (Z11-16:OH), and (*Z*)-11-hexadecenyl acetate (Z11-16:Ac)^17^,^18^,^19^,^20^,^21^,^22^. Behaviour experiments in *H. armigera* have proven that Z11-16:OH and 16:OH can act as inhibitors when mixed with attractive blends^19^, whereas Z9-14:Ald is an agonist when presents at low concentrations in the attraction blends and an antagonist at high concentrations^23^,^24^. In *H. assulta*, field tests have reported that small amounts of Z9-14:Ald or Z9-16:OH added to the main pheromone blend significantly reduce the caught of male *H. assulta*^17^,^25^.

Electrophysiological recordings have been applied to the study of peripheral coding of sex pheromones in *Heliothis/Helicoverpa* species. In *Heliothis zea*, *Heliothis virescens* and *Heliothis subflexa*, three basic types of pheromone-responsive sensilla named type A, B and C, with different population in the male antenna have been reported^26^,^27^. The most represented sensilla, type A, housed two neurons, one tuned to the major pheromone component (Z11-16:Ald), the other to still unknown stimuli. Sensilla of type-B also contained two neurons, the first responding to Z9–14:Ald (and also to Z9-16:Ald in *H. subflexa*), the second to still unidentified volatiles. The two neurons housed in sensilla of type C were less specific and responded to a number of pheromone components with marked differences between the three species. In *H. zea*, the C-type sensilla contained one large-spike-amplitude neuron tuned to two pheromone components Z9-16:Ald and Z9-14:Ald and one smaller -spike-amplitude neuron tuned to three known behavioral antagonists Z9-14:Ald, Z11-16:Ac, and Z11-16:OH. In *H. virescen*s, the neuron specific for Z9–14:Ald was colocalized with the one specific for two behavioral antagonists Z11–16:OH and Z11–16:Ac. In *H. subflexa*, the neuron responding only to Z11-16:OH was colocalised with the neuron responding to Z11-16:Ac and Z9-14:Ald. In a previous study on *H. armigera* and *H. assulta*, instead Wu *et al.* (2013) identified two types of sensilla trichodea which were specifically tuned to the major pheromone components Z11-16:Ald and Z9-16:Ald respectively. Interestingly, the ratios of Z11-16:Ald- and Z9-16:Ald sensitivity OSNs were 100:28.9 and 21.9:100 in *H. armigera* and *H. assulta* respectively, well reflecting the relative ratios of the major pheromone components in the two species^28^,^29^.

The function of PRs had been studied in *Noctuidae* moths especially in *H. virescens*. Six sex pheromone receptor (PR) candidates in *H. virescens* were identified by using a combination of genomic sequence analysis, cDNA-library screening as well as BAC library sequence. Functional characterization of PRs was also performed in heterologous expression systems, such as *Xenopus* oocytes or HEK293 cell culture. The receptors’ functional activity was closely associated with pheromone-sensitive neuronal function from single sensilla recordings. HvOR13 in A-type sensilla and HvOR6 in B-type sensilla specifically tuned to Z11-16:Ald, the major pheromone component and Z9-14:Ald, the second component, respectively. HvOR14 and HvOR16 in C-type sensillum were, respectively, tuned to Z11-16:Ac and Z11-16:OH. Also in our previous work, we sequenced and analyzed the antennal transcriptome of *H. armigera* and *H. assulta*. Seven *H. armigera* pheromone receptor genes (HarmOR6, HarmOR11, HarmOR13, HarmOR14, HarmOR14b, HarmOR15 and HarmOR16) and six from *H. assulta* (HassOR6, HassOR11, HassOR13, HassOR14, HassOR14b and HassOR16) had been identified^30^,^31^. Six of the HarmPRs have also been functionally characterised by heterologous expression in *Xenopus* oocytes in our previous work. HarmOR13 was found to be a specific receptor for the major sex pheromone component Z11-16:Ald, HarmOR6 was equally tuned to both Z9-16:Ald and Z9-14:Ald, while HarmOR16 was sensitive to Z11-16:OH. HarmOR11, HarmOR14 and HarmOR15, instead, failed to respond to any pheromone compounds^32^. Other studies have recently reported that HarmOR6 is highly tuned to Z9-16:OH, a pheromone analogue we did not test in our previous work, as well as to the two pheromone components Z9-16:Ald and Z9-14:Ald. This behaviour has also been recently confirmed in our lab. These experiments elucidated the functions of some pheromone receptor genes of *H. armigera*, as a contribution for understanding intraspecific mating choice and speciation extension in moths at the molecular level.

In this work, we have applied single sensillum recording to map the different functional types of sensilla trichodea on the male antennae of the two sibling species *H. armigera* and *H. assulta* and used two-colour *in situ* hybridization technique to identify which ORs are expressed in each type of sensilla.

## Results

### Types of sensilla on the antennae of *H. armigera* and *H. assulta*

**Figure 1:**
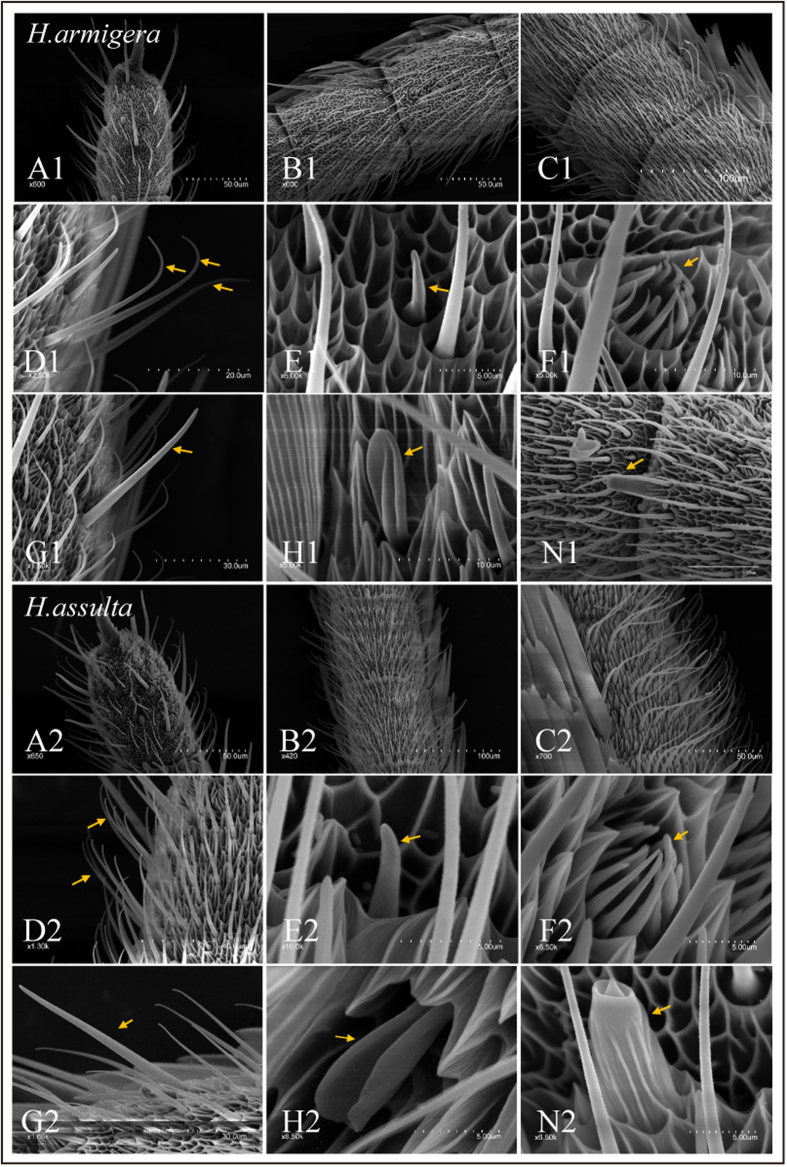
Scanning electron micrographs of sensilla on male *H. assulta* and *H. armigera* antenna. Gross morphology of the distal (**A**), middle (**B**) and proximal (**C**) region of the male antenna. Scanning electron microscopy (SEM) revealed at least six morphological types of sensilla along male *H. assulta* and *H. armigera* antennae, including sensilla trichoid (**D**), basiconica (**E**), coeloconica (**F**), chaetica (**G**), auricillica (**H**) and styloconica (**N**), indicated by an arrow in each image. Scale bar: C1, B2: 100 μm; A1, B1, A2, C2: 50 μm; D2:40 μm; G1, G2: 30 μm; D1: 20 μm; F1, H1, N1: 10 μm; E1, E2, F2, H2, N2: 5 μm.

We first performed a morphological analysis of the sensilla detected on the antennae of *H. armigera* and *H. assulta*, using Scanning Electron Microscopy (SEM). As in other Lepidopteran species, we detected six types of sensilla, trichoid, basiconica, coeloconica, chaetica, auricillica and styloconica, according to the classification systems described in the literatures^33^,^34^. Figure 1 reports some representative images for each type of sensory hair. Most of sensilla were located on the ventral surface, while the dorsal side was covered with scales. As shown in Fig. 1, the apical, middle and basal segments of *H.armigera* (A1-C1) and *H.assulta* (A2-C2) exhibit similar external morphology and distribution of sensilla. Sensilla thrichodea (D1, D2) were found to be the most abundant, representing about 80% of total. Both the total number of sensilla and their relative distributions were not significantly different between the two species (Supplementary Table 1). We could also observe that the total number of hairs progressively decreased from the base to the tip of the antenna (Fig. 1A–C).

### Functional characteristics of OSNs in sensilla trichoidea responding to sex pheromone components

In order to identify and classify different functional types of sensilla trichodea, we performed single-sensillum recording from 839 of such sensilla in 86 male antennae of *H. armigera* and 680 from 71 antennae of *H. assulta*.

As stimuli, we used most of the pheromone components as identified in the female pheromone glands of *H. armigera* and *H. assulta*, namely Z11-16:Ald, Z9-16:Ald, Z9-14:Ald, Z9-16:OH, Z11-16:OH and Z11-16:Ac, with the addition of two analogues, Z9-14:Ac and Z9-14:OH. These two compounds which have been identified in the female pheromone glands of *Spodoptera exigua* have been added to the list to verify whether *H. armigera* and *H. assulta* could recognise and discriminate their pheromone components from those of other species.

Based on the selectivity of electrophysiological responses, we detected three types of trichod sensilla in *H. armigera*, A, B and C, but only two, A and C, in *H. assulta*. In *H. armigera*, the majority of sensilla (445, 53%) are of type A, while those of type B (17, 2%) and C (86, 10.3%) are much less abundant. In *H. assulta*, types A and C are equally represented (182, 26.8% and 210, 30.9%, respectively), but we could not detected sensilla of type B among the 680 examined. In addition, we could not record any activity from 42.4% (288 out of 680) of sensilla in *H. assulta* and 34.7% (291 out of 839) in *H. armigera*. These sensilla also showed spontaneous activity and most of them contained two OSNs.

**Figure 2:**
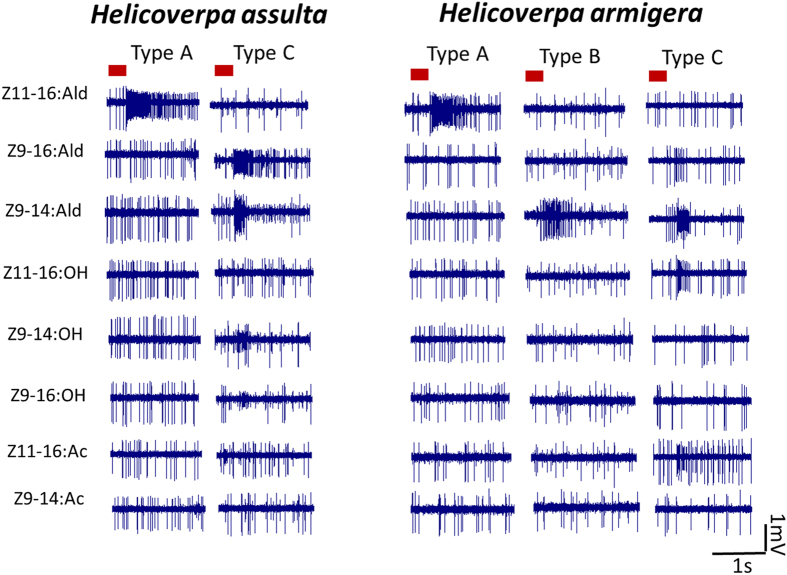
Single-sensillum recordings from antennal trichoid sensilla. Stimuli are Z11-16:Ald, Z9-16:Ald, Z9-14:Ald, Z11-16:OH, Z9-14:OH, Z9-16:OH, Z11-16:Ac and Z9-14:Ac. The red bold line represents 0.3 s odour stimulation. The amount of each stimuli was 1 mg. We detected three functional types of trichod sensilla in *H. armigera*, (**A**–**C**) but only two, (**A**,**C**) in *H. assulta*. In *H. armigera*, type (**A**,**B**) were specifically tuned to Z11-16:Ald and Z9-14:Ald, respectively, while type (**C**) showed a broad response to Z9-14:Ald, Z9-16:Ald, Z11-16:OH and Z11-16:Ac. In *H. assulta*, type A was specific for to Z11-16:Ald, while type (**C**) was broadly tuned to Z9-14:Ald, Z9-16:Ald, Z9-14:OH and Z9-16:OH.

**Figure 3:**
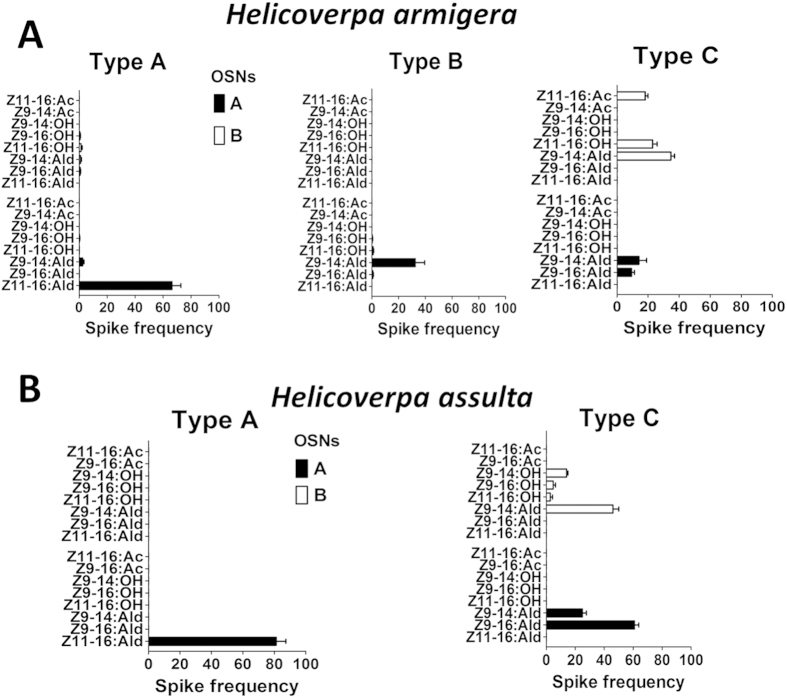
Response profiles of two distinct OSNs (A,B) housed together in different types of sensilla on the antenna of male *H. armigera* (A) and *H. assulta* (B) to 1 mg stimuli. The response value (Spike frequency) was calculated as the difference in spike number recorded 1 s before and 1 s after the stimulus delivery. Error bars indicate SEM (n = 7–13).

Based on different spike amplitudes, we found that all functional types of sensilla in both species house two distinct OSNs. In sensilla of type A, OSN-A exhibited in both species very selective and strong response to the same compound, Z11-16:Ald, which is the major pheromone component of *H. armigera*, while we could not record any response from OSN-B using our set of stimuli. Similar behaviour was observed in sensilla of type B in *H. armigera*, with one OSN responding only to Z9-14:Ald, while the other was not sensitive to any of the tested compounds. Sensilla of type C were less specific in both species. OSN-A responded only to Z9-14:Ald and Z9-16:Ald. In particular, the sensitivity for the latter compound, which is the major pheromone component in *H. assulta*, was much higher in this species. OSN-B in the same type of sensilla also responded to 3–4 compounds with different patterns between the two species (Figs 2 and 3).

**Figure 4:**
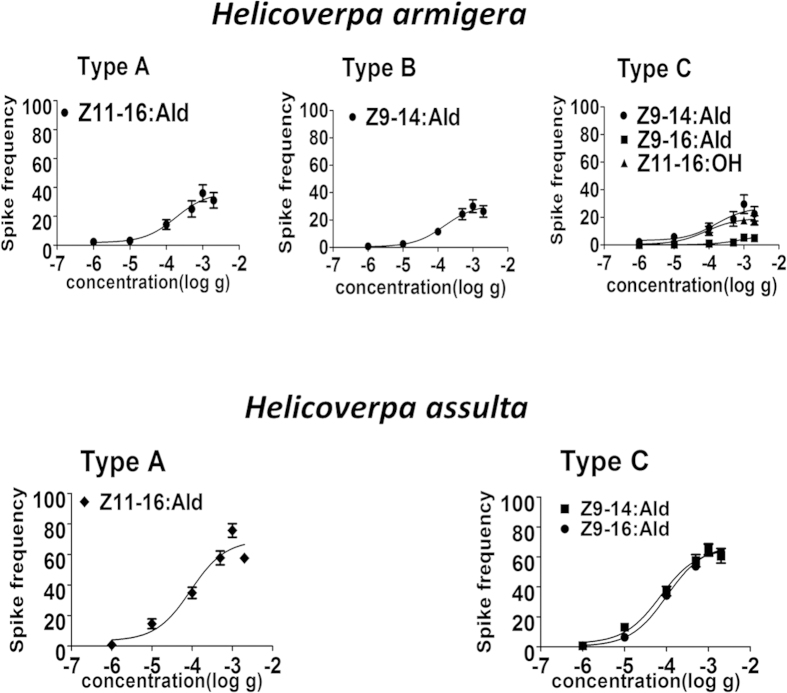
Concentration-response relationships of OSNs housed in different physiological types of trichoid sensilla on the antennae of male *H. armigera* and *H. assulta*. (**A**) Dose-response curves of OSNs housed in sensilla of type (**A**–**C**) on the antennae of male *H. armigera*. Spike frequency was calculated as the difference in spike number recorded 1 s before and 1 s after the stimulus delivery. Error bars indicate SEM (n = 7–13). (**B**) Dose-response curves of OSNs housed in sensilla of type (**A**,**C**) on the antennae of male *H. assulta*. Spike frequency was calculated as the difference in spike number recorded 1 s before and 1 s after the stimulus delivery. Error bars indicate SEM (n = 10–13).

We next measured the responses of the same sensilla to their active stimuli across a dose range from 1 µg to 2 mg (Fig. 4). All the compounds showed sigmoidal dose-response curves with half-maximum values around 100 µg and threshold about 10 to 100 times lower. Such values were compatible with the sensitivity of the insect antenna, as reported in other studies, considering that our samples were dissolved in paraffin oil, a very good solvent for such molecules, thus strongly reducing the fraction of pheromone presented in the air. We could also observe that for all the chemicals tested, the sensitivity of *H. assulta* was about twice that of *H. armigera*.

### Distribution of functional trichoid sensilla along the antenna

**Figure 5:**
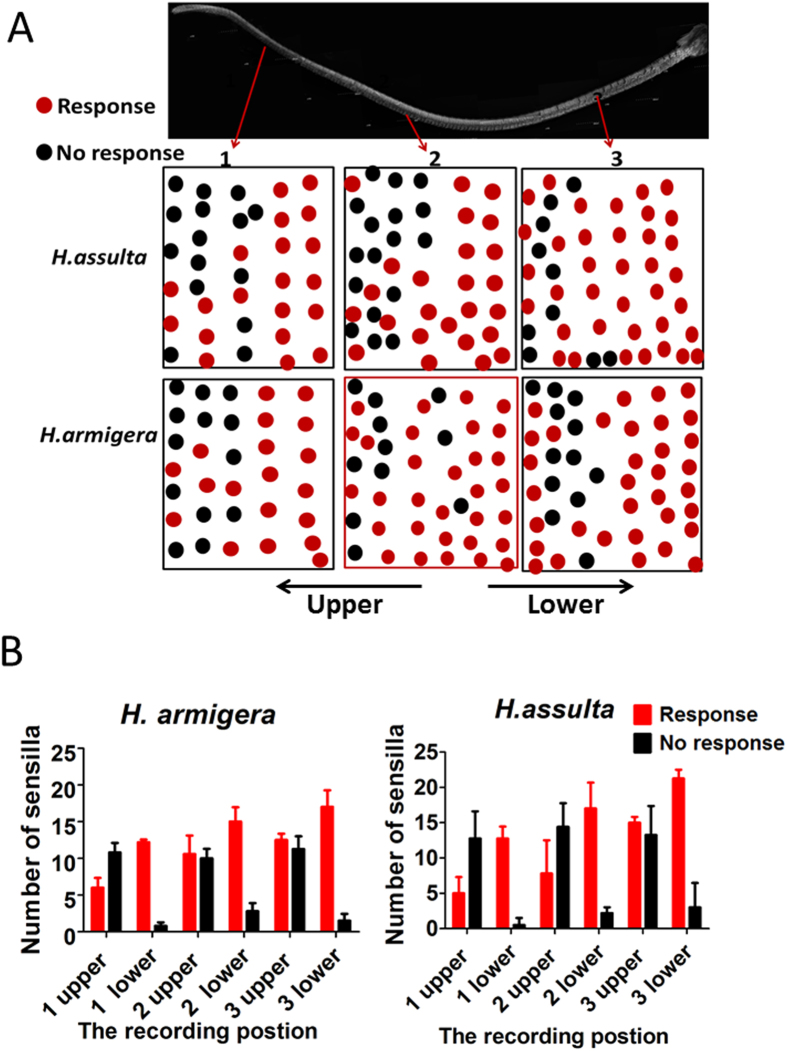
Distribution of functional and non-functional trichoid sensilla on the antennae of male *H. armigera* and *H. assulta*. (**A**) Map of functional and non-functional trichoid sensilla on the antennae of male *H. armigera* and *H. assulta* (**A**). The whole antenna was divided into three parts, containing annuli 1–20, 21–50 and 51–70 Black circles indicate trichoid sensilla which did not respond to any of the stimuli applied. Red circles rounds indicate trichoid sensilla with responses to one or more ligands. (**B**) Percentages of functional and non-functional trichoid sensilla on the antennae of male *H. assulta* and *H. armigera*. Each annulus is divided in the middle, the left part being the distal and the right part the proximal region. Error bars indicate SD (n = 5).

When we looked at the spatial distribution of functional sensilla on the antenna, we could not detect any clustering of sensilla with the same specificity of response. However, we observed that functional trichoid sensilla were mostly located in the proximal region of each annulus, while inactive sensilla were generally found close to the distal end. Such arrangement was verified in both *H. assulta* and *H. armigera* (Fig. 5).

### The intriguing case of sensillum type B

**Figure 6:**
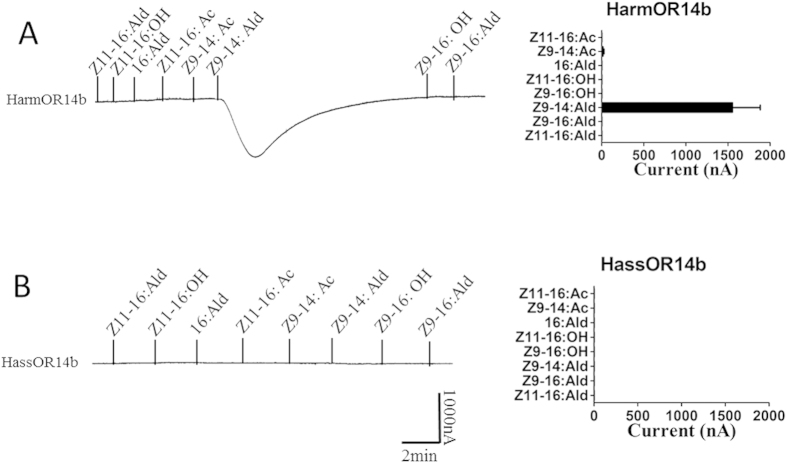
Functional analysis of HarmOR14b (A) and *HassOR14b* (B) genes in *Xenopus* oocytes. In each panel: (Left) Inward current responses of HarmOR14b/HarmOrco and HassOR14b/HassOrco-coexpressed in *Xenopus* oocytes to 10^−4^ mol/L sex pheromone components and analogs. (Right) Response profiles of HarmOR14b and HassOR14b. Error bars indicate SEM (n = 6).

The specific occurrence of trichoid sensilla of type B only in *H. armigera* poses an interesting question on its role in pheromone detection and species segregation. The case is even more intriguing for the fact that this sensillum only responds to Z9-14:Ald, a chemical which is also found in the gland secretion of female *H. armigera*. Therefore, based on previous functional studies of olfactory receptors (ORs) in *H. armigera*, performed in our lab^32^, we decided to characterize the response of OR14b in both species. In *H. armigera*, this receptor, when expressed in *Xenopus* oocytes, responds specifically to Z9-14:Ald (Fig. 6). However, its *H. assulta* orthologue does not respond to any of the compounds tested, despite a very high sequence similarity between the two amino acid sequences (406/440 = 92% identity), with only 5 amino acids substitutions occurring in the external loops (Supplementary Figure 2).

### Response characteristics of pheromone receptors

Except for surveying the response profiles of OR14b in the two species, we also examined the response profiles of four other PRs (HassOR6, HassOR11, HassOR13 and HassOR16) to eight pheromone ligands in *H. assulta*, using two electrode voltage clamp recordings. The results showed that HassOR13/Orco and HassOR16/Orco were narrowly tuned to Z11-16:Ald and Z9-14:Ald respectively, while HassOR6/Orco responded mainly to Z9-16:OH, but also to several other components including Z9-16:Ald and Z9-14:Ald. HassOR11/Orco still did not respond to any of the ligands tested (Supplementary Figure 1). We have not cloned the full length sequence of HassOR14 until now, therefore we could not perform any functional study with this gene. All the results obtained so far are consisted with the previous data^29^,^32^.

### Olfactory receptors expressed in neighboring OSNs in the same trichoid sensilla

**Figure 7:**
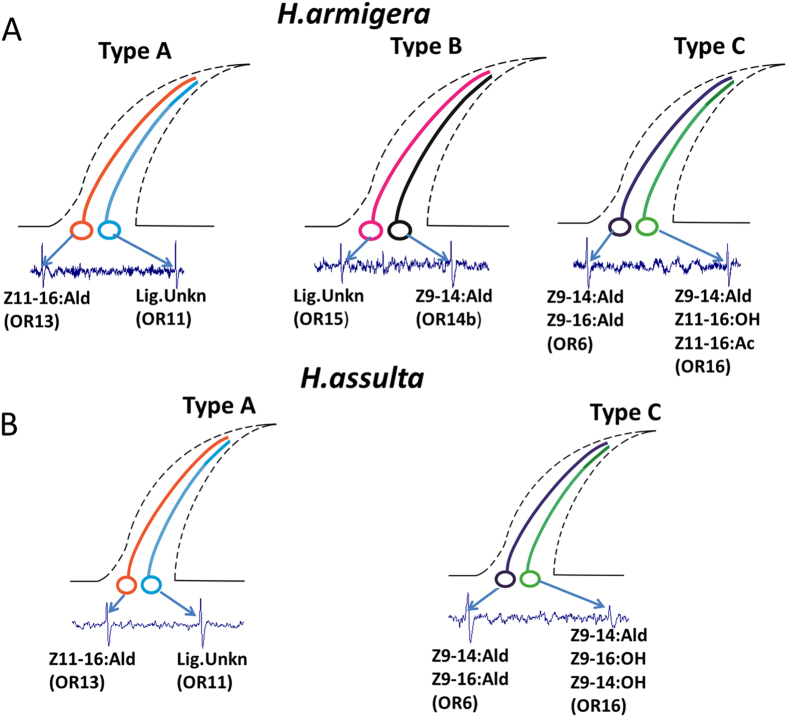
Model of sensillar compartmentalization arrangements in type-A, -B and -C sensilla of male *H. armigera* (A) and *H. assulta* (B).

**Figure 8:**
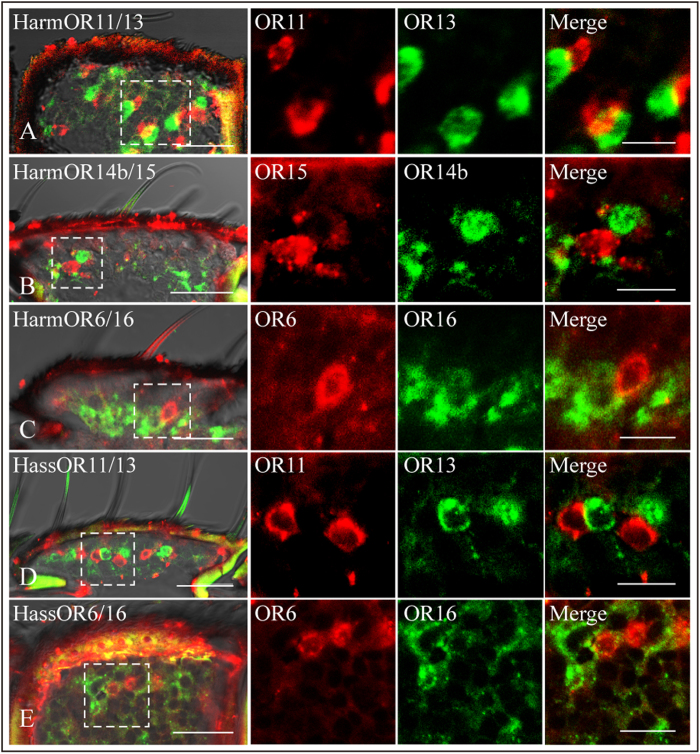
Two-colour *in situ* hybridization visualizing the combinations of HarmOR11/HarmOR13 (A), HarmOR14b/HarmOR15 (B), HarmOR6/HarmOR16 (C), HassOR11/HassOR13 (D) and HassOR6/HassOR16 (E) in a proximal segment of male antenna. In each panel, the left image shows an overlay of optical planes from a stack of optical sections. Higher magnification of the boxed area is also shown in the right image. The two middle images show a single fluorescence channel of optical planes from a stack of optical sections. (Scale bar: A-E: 20 μm; the boxed area: 10 μm).

Combining the information obtained by single-sensillum recording on the specificities of each sensillum to chemical stimuli with that collected on the responses of individual ORs to the same chemical compounds, we could predict the location of specific ORs in each type of sensilla. In the A type sensilla of *H. assulta* and *H. armigera*, OR13, responding specifically to Z11-16:Ald, was supposed to be expressed on the smaller-spike-amplitude neuron tuned to Z11-16:Ald, while an OR for which no ligand had been found, was predicted to be expressed in the larger-spike-amplitude unresponsive neuron (Fig. 7). Based on the published evidence that in male *H. virescens*, HR11 and HR13 are located in two adjacent cells^35^, we could predict that a similar pattern could also exist in A type sensilla in *H. assulta* and *H. armigera*. This hypothesis was verified with the two-colour *in situ* hybridization experiments of OR11 and OR13. The strong signals of OR11 and OR13 were found expressed side by side with some overlapping in male antenna of the two species (Fig. 8A,D). In type C sensilla of both *H. assulta* and *H. armigera*, OR6, which responds to Z9-16:OH, Z9-14:Ald and Z9-16:Ald, matched with the larger-spike-amplitude OSN tuned to Z9-14:Ald and Z9-16:Ald. HarmOR16 tuned to Z9-14:Ald and Z11-16:OH and HassOR16 mainly responding to Z9-14:Ald matched with the smaller-spike-amplitude OSN responding to Z9-14:Ald and Z11-16:OH in *H. armigera* and to Z9-14:Ald in *H. assulta* (Fig. 7). The expression of OR6 and OR16 in one sensillum was also demonstrated by *in situ* hybridization. The side by side signal of OR6/OR16 could be found in different segments of antennae, although it was much weaker than that of OR11/OR13 (Fig. 8C,E). In type B sensilla of *H. armigera*, HarmOR14b, which responds to Z9-14:Ald, matched with the larger-spike-amplitude OSN tuned to Z9-14:Ald, while an OR for which no ligand had been found, was predicted to be expressed in the smaller-spike-amplitude unresponsive neuron (Fig. 7). The results of two color *in situ* hybridization proved that HarmOR15 and HarmOR14b were located in two adjacent cells of the same sensillum (Fig. 8B).

In summary, we found colocalisation of OR13 and OR11 in sensilla of type A in both species, of OR6 and OR16 in sensilla of type C in both species, and of OR14b and OR15 in sensilla of type B in *H. armigera*. These predicted combinations are summarized in Fig. 7.

## Discussion

Aim of this work was to understand how peripheral coding contributes to different behaviour in the two species *H. armigera* and *H. assulta* using the same pheromone components in different ratios. The phenomenon of two closely related lepidopteran species using the same pheromone components in different ratios is not limited to *Helicoverpa*, but the mechanism of peripheral coding of pheromone might not be the same. It has been reported that the response profile of the sex pheromone receptors^36^, or their expression pattern^37^, can directly account for the attractive behaviour in silk moths. In the genus *Ostrinia*, a single amino acid polymorphism in OR3 provides a major shift in the specificity for the sex pheromone components of two closely related species, the Asian and the European corn borers (ACB and ECB)^38^. In this case it is the response profiles of the pheromone receptors, rather than their expression pattern, responsible for the phenomenon observed in *Ostrinia* species. In *H. armigera* and *H. assulta*, instead, the mechanism of peripheral coding differentiation is more complex, due to differences both in the function and expression of pheromone receptors. Previous work had revealed the presence of seven genes encoding PRs in *H. armigera* and six in *H. assulta*^30^,^31^. OR13 and OR6 orthologs of these two species are similar in function, as they are tuned to the two major pheromone components Z11-16:Ald and Z9-16:Ald respectively. Therefore, the attraction of *H. armigera* and *H. assulta* to reverse ratios of the two components mainly depend on the relative expression levels the two ORs^29^,^32^. Meanwhile, the receptors for the minor pheromone components exhibited different functions. Although OR14b and OR16 orthologs also share high amino acid identities, the functions are different. OR14b in *H. armigera* was tuned to Z9-14:Ald but did not show any response in *H. assulta*. OR16 in *H. armigera* was tuned to two behavioural antagonists Z9-14:Ald and Z11-16:OH, while in *H. assulta* it was specifically tuned to Z9-14:Ald. It seemed that during the evolution of *H. assulta* and *H. armigera*, most of similarity receptors keep function consistency, but it can’t be ignore that the speciation events could lead to the function divergence of some PR orthologs.

Based on such information, we have mapped the distribution of neurons expressing different PRs in trichoid sensilla and how these were clustered on the antenna (Fig. 7). The difference in the number and function of sensilla trichodea in these two species were consistent with the function and expression differentiation of pheromone receptors.

In *H. armigera*, most of sensilla trichodea (type A) housed two neurons: one expressing OR13 tuned to the major pheromone component Z11-16:Ald, the other expressing OR11, so far not responding to any pheromone component and suggested to be tuned to general odorants or perhaps to breakdown products of Z11-16:Ald. However Lee (2006) reported that the neuron expressing OR11 in *H. subflexa* did not respond to any of more than 60 compounds including several general odorants, as well as some *Heliothine* sex pheromone components and some breakdown products^39^. Sensilla thrichodea of type B are much less abundant than type A and contain two neurons, expressing OR14b and OR15. The first is tuned to Z9-14:Ald, while the second did not respond to any of the tested compounds. This fact could explain the observation that Z9-14:Ald can reproduce the effect of the natural minor component Z9-16:Ald in the pheromonal blend by enhancing the attraction of *H. armigera* males.

The two neurons housed in the third type of sensilla trichodea (C-type) express OR6 and OR16, the first tuned to agonists (Z9-16:Ald and Z9-14:Ald), the second to antagonists (Z9-14:Ald, Z11-16:OH and Z11-16:Ac). It is interesting to observe that Z9-14:Ald can act both as an agonist and an antagonist, depending on the neuron it activates. In fact, at low concentrations it stimulates the same neuron tuned to the second sex pheromone component Z9-16:Ald, linked to an attractive behaviour. At high concentration, instead, it can switch-on the less sensitive OR16, producing an opposite behaviour.

In the two neurons of type C sensilla of *H. assulta* we find the two *H. armigera* orthologue ORs, tuned to the same chemicals. The first (OR6) is linked to an agonist behaviour, as Z9-16:Ald is the major sex pheromone component in this species, the second is again tuned to antagonists. Apart from these three types of active sensilla, we also found a large number of other sensilla trichodea which did not respond to any of the chemicals tested. The question remains whether such sensilla might be tuned to general odorants or else they house some non-functional ORs.

Regarding the spatial distribution of these types of sensilla, it was interesting to find that functional and non-functional trichoid sensilla were clustered in the proximal and distal regions of each annulus, respectively. Previous studies have also reported some arrangement of functionally different sensilla in *Drosophila* and in *Manduca sexta*^40^,^41^,^42^. Such spatial organization has been proposed to be convenient for discriminating different pheromone components and certainly produces a simpler wiring of the axons between the periphery and the glomeruli of the antennal lobes.

## Methods

### Insects

*H. armigera* and *H. assulta* were reared at the Institute of Plant Protection, Chinese Academy of Agricultural Sciences, Beijing, China. Larvae were reared on an artificial diet and placed on a 16:8 h (light: dark) photoperiod at 25 ± 1 °C, 55–65% RH. Pupae were sexed and male and female individuals were placed in separate cages for eclosion. The adults were fed on 10% honey solution. One-to-four-day-old virgin adults were used in all experiments.

### Pheromone components

The nine pheromone components (Z11-16:Ald, Z9-16:Ald, Z9-14:Ald, Z11-16:OH, Z9-16:OH, Z9-14:OH, Z11-16:Ac, Z9-14:Ac and 16Ald, all 96% minimum purity) used in this study were obtained from Nimrod Inc (Changzhou, China). For SSR experiment, the pheromone components were dissolved in paraffin oil at the concentration of 100 μg/μl and stored at −20 °C. For voltage-clamp electrophysiological recordings, pheromone components were dissolved in dimethyl sulfoxide (DMSO) at 1 M concentration and stored at −20 °C.

### Scanning electron microscopy

Antennae were dissected from one-day old male moths and transferred to 2.5% glutaraldehyde solution, then fixed for 2 hours at 4 °C. After three washes at room temperature with 0.1 M PBS, antennae were incubated for 2 hours at 4 °C, then washed three times with 0.1 M PBS. Samples were dehydrated using an ethanol series (30%, 50%, 70%, 80% and 90%), followed by three washings in100% ethanol. After drying in a critical point drier (LEICA EM CPD), antennae were sprayed with gold (EIKO IB-3). Finally, samples were observed in a Hitachi S-3400N scanning electron microscope.

### Single sensillum recording

Male adults were restrained in a 1-ml plastic pipette tip with the whole head protruding, fixed to the rim of the pipette tip with dental wax, while one of the exposed antenna was attached to a coverslip with double-face adhesive tape.

For recording, we used a tungsten wire inserted into the eye of moth as a reference electrode. The recording electrode was also a tungsten wire, which was electrolytically sharpened by repeatedly immersing the tip into 28% KNO_2_ solution. The recording electrode was attached to an olfactory probe (Syntech) and inserted into the base of each sensillum trichodeum until a stable electrical signal with a high signal-to-noise ratio was attained. The recordings were performed under a LEICA Z16 APO microscope at 920 × magnification.

For stimulus delivery, 10 μl of the solution was dripped on a filter paper strip (0.8 cm × 2.6 cm) inserted in a Pasteur pipette (15 cm long). A flow of purified and humidified air continuously blew toward the antenna through a 14-cm-long metal tube controller (Syntech, Hilversum, Netherlands). The fixed antennae were exposed to a 300-ms odour air pulse with an air flow of 20 ml/s delivered through a Pasteur pipette. The pre-amplifier was set at a gain of 10×. Using the software package Autospike (Syntech), action potentials were amplified, digitized and visualized on a computer screen. The number of OSNs housed in single sensillum could be defined based on their differences in spike amplitudes. The response values to specific odour stimuli were calculated as the differences in spike numbers observed between 1 second before and 1 second after stimulus delivery.

To map the distribution of different functional types of sensilla trichoidea in the antennal flagella, we divided the whole antenna, comprising 70 annuli, into three parts (1–20, 21–50, 51–70). In each part, we selected only one segment from which to record.

### Receptor expression *in vitro* and electrophysiological recordings

The ORFs of encoding HarmOR14b, HassOR6, HassOR11, HassOR13, HassOR14b and HassOR16 were amplified and cloned into pT7Ts vector. The cRNAs of all ORs were synthesized using mMESSAGE mMACHINE T7 kit (Ambion, Austin, TX). Two-electrode voltage-clamp electrophysiological recordings were performed along with the procedure previously reported^32^,^43^,^44^.

### *In Situ* Hybridization

Antennae of male moths were embedded with NEG 50^TM^ compound (Thermo Fisher, Cheshire, UK) in Tissue-Tek Cryomold (CITOTEST, HaiMen, China) and frozen at −60 °C rapidly. Longitudinal sections (12 μm) of antennae were sectioned with cryostat microtome (LEICA CM1850) at −23 °C and pasted on Superfrost Plus slides (Thermo Fisher, New Hampshire, U.S.A.), then air-dried at room temperature for about 20 min.

DIG-labeled or Biotin-labeled antisense probes were synthesized with T7/SP6 DIG/Biotin RNA Labeling system (ROCHE, Mannheim, Germany). Two-colour fluorescence *in situ* hybridization was performed as described in references^45^,^46^ DIG-labeled RNA probes were detected by anti-DIG alkaline phosphatase-conjugated antibody (ROCHE) and visualized by the using HNPP Fluorescent Detection Set (ROCHE). For biotin-labeled RNA probes, the tyramide-signal amplification (TSA) kit (PerkinElmer, Boston, U.S.A.) was used. Sections were mounted using SlowFade Gold Antifade reagent (Molecular Probes, CA, U.S.A.). The slides were sealed with nail polish. Finally, the labeled signals were imaged and analyzed by Zeiss LSM780 laser scanning confocal microscope. Images were adjusted and arranged with Photoshop CS6 (Adobe) and Illustrator CS5 (Adobe). The contents of images were not modified except for the brightness or contrast.

## Additional Information

**How to cite this article**: Chang, H. *et al.* Sensillar expression and responses of olfactory receptors reveal different peripheral coding in two *Helicoverpa* species using the same pheromone components. *Sci. Rep.* **6**, 18742; doi: 10.1038/srep18742 (2016).

## Supplementary Material

Supplementary Information

## References

[b1] Hansson B. S. Olfaction in Lepidoptera. Experientia. 51, 1003–1027 (1995).

[b2] Hildebrand J. G. & Shepherd G. M. Mechanisms of olfactory discrimination: converging evidence for common principles across phyla. Annu. Rev. Neurosci. 20, 595–631 (1997). 9056726 10.1146/annurev.neuro.20.1.595

[b3] Hallem E. A., Dahanukar A. & Carlson J. R. Insect odor and taste receptors. Annu. Rev. Entomol. 51, 113–135 (2006). 16332206 10.1146/annurev.ento.51.051705.113646

[b4] Kaissling K. E., Zack S. & Rumbo E. R. Adaptation processes in insect olfactory receptors: mechanisms and behavioral significance. Ann. N. Y. Acad. Sci. 510, 104–112 (1987). 3324874 10.1111/j.1749-6632.1987.tb43475.x

[b5] Kaissling K. E. Physiology of pheromone reception in insects (an example of moths). Anir. 6, 73–91 (2004).

[b6] Heinbockel T. & Kaissling K. E. Variability of olfactory receptor neuron responses of female silkmoths (*Bombyx mori L*.) to benzoic acid and (6)-linalool. J. Insect. Physiol. 42, 565–578 (1996).

[b7] Den Otter C. J. Single sensillum responses in the male moth *Adoxophyes orana* (F.v.R.) to female sex pheromone components and their geometrical isomers. J. Comp. Physiol. 121, 205–222 (1977).

[b8] van der Pers J. N. C., Tomas G. & Den Otter C. J. Interactions between plant odours and pheromone reception in small ermine moths (Lepidoptera: Yponomeutidae). Chem. Senses. 5, 367–371 (1980).

[b9] Ochieng S. A., Park K. C. & Baker T. C. Host plant volatiles synergize responses of sex pheromone-specific olfactory receptor neurons in male *Helicoverpa zea*. J. Comp. Physiol. A. 188, 325–333 (2002). 10.1007/s00359-002-0308-8 12012103

[b10] Leal W. S. Odorant reception in insects: roles of receptors, binding proteins, and degrading enzymes. Annu. Rev. Entomol. 58, 373–391 (2013). 23020622 10.1146/annurev-ento-120811-153635

[b11] Touhara K. & Vosshall L. B. Sensing odorants and pheromones with chemosensory receptors. Annu. Rev. Physiol. 71, 307–332 (2009). 19575682 10.1146/annurev.physiol.010908.163209

[b12] Fitt G. P. The ecology of *Heliothis* species in relation to agroecosystems. Annu. Rev. Entomol. 34, 17–53 (1989).

[b13] Mitter C., Poole R. W. & Matthews M. Biosystematics of the *Heliothinae* (Lepidoptera, Noctuidae). Annu. Rev. Entomol. 38, 207–225 (1993).

[b14] Wang C. Z. & Dong J. F. Interspecific hybridization of *Helicoverpa armigera* and *H. assulta* (Lepidoptera: Noctuidae). Chinese. Sci. Bull. 46, 489–491 (2001).

[b15] Zhao X. C. *et al.* Hybridization between *Helicoverpa armigera* and *Helicoverpa assulta*: development and morphological characterization of F1 hybrids. B. Entomol. Res. 95, 409–416 (2005). 10.1079/ber2005372 16197561

[b16] Nesbitt B. F., Beevor P. S., Hall D. R. & Lester R. Female sex pheromone components of the cotton bollworm, Heliothis armigera. J. Insect. Physiol. 25, 535–541 (1979).

[b17] Cork A. *et al.* Female sex pheromone of *Helicoverpa assulta*(Guenee) (Lepidoptera: Noctuidae); identification and field testing. J. Chem. Ecol. 18, 403–418 (1992). 24254945 10.1007/BF00994240

[b18] Kehat M. & Dunkelblum E. Behavioral responses of male *Heliothis armigera* (Lepidoptera: Noctuidae) moths in a flight tunnel to combinations of components identified from female sex pheromone glands. J. Insect. Behav. 3, 75–83 (1990).

[b19] Wu D. M., Yan Y. H. & Cui J. R. Sex pheromone components of *Helicoverpa armigera*: chemical analysis and field tests. Entomologia. Sinica. 4, 350–356 (1997).

[b20] Zhang J. P., Salcedo C., Fang Y. L., Zhang R. J. & Zhang Z. N. An overlooked component: (Z)-9-tetradecenal as a sex pheromone in *Helicoverpa armigera*. J. Insect. Physiol. 58, 1209–1216 (2012). 22732233 10.1016/j.jinsphys.2012.05.018

[b21] Kehat M., Gothilf S., Dunkelblum E. & Greenberg S. Field evaluation of female sex pheromone components of the cotton bollworm, Heliothis armigera. Entomol. Exp. Appl. 27, 188–193 (1980).

[b22] Liu M. Y., Cai J. P. & Tian Y. Sex pheromone components of the oriental tobacco budworm, *Helicoverpa assulta* Guenée:identification and field trials. Entomol. Sinica. 1, 77–85 (1994).

[b23] Rothschild G. Attractants fo *Heliothis armigera* and *Heliothis punctigera*. J. Aust. Entomol. Soc. 17, 389–390 (1978).

[b24] Gothilf S., K M., Jacobson M. & Galun R. Sex attractants for male *Heliothis armigera* (Hbn.). Cell. Mol. Life. Sci. 34, 853–854 (1978).

[b25] Boo K. S. *et al.* (Z)-9-tetradecenal: a potent inhibitor of pheromone-mediated communication in the oriental tobacco budworm moth;*Helicoverpa assulta*. J. Comp. Physiol. A. 177, 695–699 (1995).

[b26] Baker T. C. *et al.* A comparison of responses from olfactory receptor neurons of *Heliothis subflexa* and *Heliothis virescens* to components of their sex pheromone. J. Comp. Physiol. A. 190, 155–165(2004). 10.1007/s00359-003-0483-2 14689220

[b27] Cossé A. A., Todd J. L. & Baker T. C. Neurons discovered in male *Helicoverpa zea* antennae that correlate with pheromone-mediated attraction and interspecific antagonism. J. Comp. Physiol. A. 182, 585–594 (1998).

[b28] Wu H., Hou C., Huang L. Q., Yan F. S. & Wang C. Z. Peripheral coding of sex pheromone blends with reverse ratios in two *Helicoverpa* species. PLoS One. 8, e70078 (2013). 23894593 10.1371/journal.pone.0070078 PMC3720945

[b29] Jiang X. J. *et al.* Sequence similarity and functional comparisons of pheromone receptor orthologs in two closely related *Helicoverpa* species. Insect. Biochem. Mol. Biol. 48, 63–74(2014). 24632377 10.1016/j.ibmb.2014.02.010

[b30] Zhang J. *et al.* Antennal transcriptome analysis and comparison of chemosensory gene families in two closely related noctuidae moths, *Helicoverpa armigera* and *H. assulta*. PLoS One. 10, e0117054 (2015). 25659090 10.1371/journal.pone.0117054 PMC4319919

[b31] Liu Y., Gu S. H., Zhang Y. J., Guo Y. Y. & Wang G. R. Candidate Olfaction Genes Identified within the *Helicoverpa armigera* Antennal Transcriptome. PLoS One. 7, e48260 (2012). 23110222 10.1371/journal.pone.0048260 PMC3482190

[b32] Liu Y., Liu C. C., Lin K. J. & Wang G. R. Functional specificity of sex pheromone receptors in the cotton bollworm *Helicoverpa armigera* PLoS One. 8, e62094 (2013). 23614018 10.1371/journal.pone.0062094 PMC3626661

[b33] Zacharuk R. Y. Ultrastructure and fuction of insect chemsensilla. Annu. Rev. Entomol. 25, 27–47 (1980).

[b34] Koh Y. H., Park K. C. & Boo K. S. Antennal sensilla in adult *Helicoverpa assulta* (Lepidoptera: noctuidae): morphology, distribution, and ultrastructure. Ann. Entomol. Soc. Am. 88, 519–530 (1995).

[b35] Krieger J. *et al.* HR11 and HR13 receptor-expressing neurons are housed together in pheromone-responsive sensilla trichodea of male *Heliothis virescens*. Chem. Senses. 34, 469–477 (2009). 19289532 10.1093/chemse/bjp012

[b36] Sakurai T. *et al.* A single sex pheromone receptor determines chemical response specificity of sexual behavior in the silkmoth *Bombyx mori*. PLoS Genet. 7, e1002115 (2011). 21738481 10.1371/journal.pgen.1002115 PMC3128102

[b37] Fujii T. *et al.* Sex-linked transcription factor involved in a shift of sex pheromone preference in the silkmoth *Bombyx mori*. Proc. Natl. Acad. Sci. USA 108, 18038–18043(2011). 22006327 10.1073/pnas.1107282108 PMC3207681

[b38] Leary G. P. *et al.* Single mutation to a sex pheromone receptor provides adaptive specificity between closely related moth species. Proc. Natl. Acad. Sci. USA 109, 14081–14086 (2012). 22891317 10.1073/pnas.1204661109 PMC3435168

[b39] Lee S. G. Pheromone-related olfactory neuronal pathways of male *Heliothine* moths [PhD thesis]. University Park, PA: The Pennsylvania State University. P, 120–166(2006).

[b40] Van der Goes van Naters W. & Carlson J. R. Receptors and neurons for fly odors in *Drosophila*. Curr. Biol. 17, 606–612 (2007). 17363256 10.1016/j.cub.2007.02.043 PMC1876700

[b41] Clyne P., Grant A., O’ Connell, R. & Carlson, J. R. Odorant response of individual sensilla on the *Drosophila* antenna. Invertebr. Neurosci. 3, 127–135 (1997). 10.1007/BF02480367 9783438

[b42] Kalinova B., Hoskovec M., Liblikas I., Vnelius C. R. & Hansson B. S. Detection of Sex pheromone Components in *Manduca sexta*. Chem. Senses. 26, 1175–1186 (2001). 11705803 10.1093/chemse/26.9.1175

[b43] Wang G. R., Carey A. F., Carlson J. R. & Zwiebel L. J. Molecular basis of odor coding in the malaria vector mosquito *Anopheles gambiae*. Proc. Natl. Acad. Sci. USA 107, 4418–4423 (2010). 20160092 10.1073/pnas.0913392107 PMC2840125

[b44] Wang G. R., Vasquez G. M., Schal C., Zwiebel L. J. & Gould F. Functional characterization of pheromone receptors in the tobacco budworm *Heliothis virescens*. Insect. Mol. Biol. 20, 125–133(2011). 20946532 10.1111/j.1365-2583.2010.01045.x

[b45] Krieger J. *et al.* A divergent gene family encoding candidate olfactory receptors of the moth *Heliothis virescens*. Eur. J. Neurosci. 16, 619–28 (2002). 12270037 10.1046/j.1460-9568.2002.02109.x

[b46] Krieger J. *et al.* Genes encoding candidate pheromone receptors in a moth (*Heliothis virescens*). Proc. Natl. Acad. Sci. USA 101, 11845–50 (2004). 15289611 10.1073/pnas.0403052101 PMC511062

