# Supplementary material for: Sensillar expression and responses of olfactory receptors reveal different peripheral coding in two Helicoverpa species using the same pheromone components
Source: Sci Rep. 2016 Jan 8;6:18742. doi: 10.1038/srep18742 (PMC4705464; doi:10.1038/srep18742)
Supplement: Supplementary Information [file srep18742-s1.pdf]

Sensillar expression and responses of olfactory receptors reveal different peripheral coding in two *Helicoverpa* species using the same pheromone components

Hetan Chang[1,2,+], Mengbo Guo[1,2,+], Bing Wang[1], Yang Liu[1,*], Shuanglin Dong[2], Guirong Wang[1,*]

Supplementary Table 1. The statistical number of different morphology sensilla on the antenna of *H. assulta* and *H. armigera*.

	Helicoverpa armigera	*Helicoverpa assulta*
sensilla trichodea	7810	8300
sensilla basiconica	800	1115
sensilla coeloconica	550	620
sensilla chaetica	486	450
Sensilla auricillica	320	290
sensilla styloconica	81	75
Total NO.	~10137	~10850

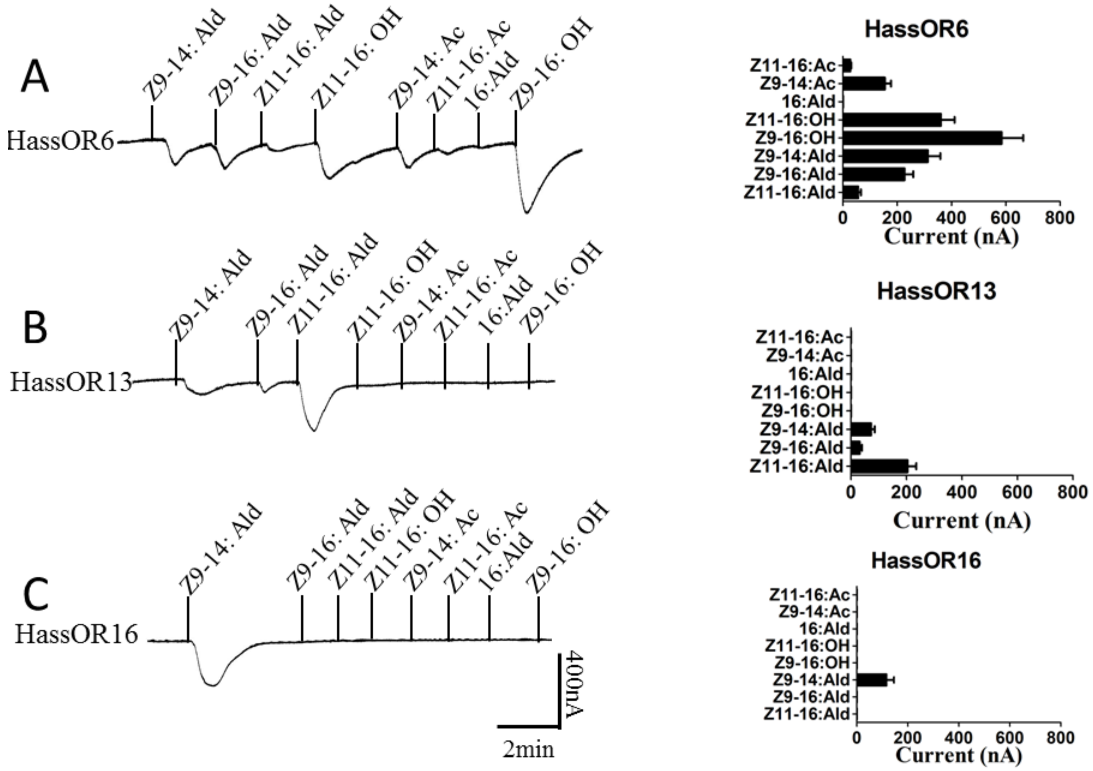

Supplementary Figure 1. Functional analysis of HassOR6 (A), HassOR13 (B) and HassOR16 (C) in *Xenopus* oocytes. In each panel: (Left) Inward current responses of HassOR/ HassOrco-coexpressed *Xenopus* oocytes to 10^{-4} mol/L sex pheromone components and analogs. (Right) Response profiles of HassORs. Error bars indicate SEM (n=10).

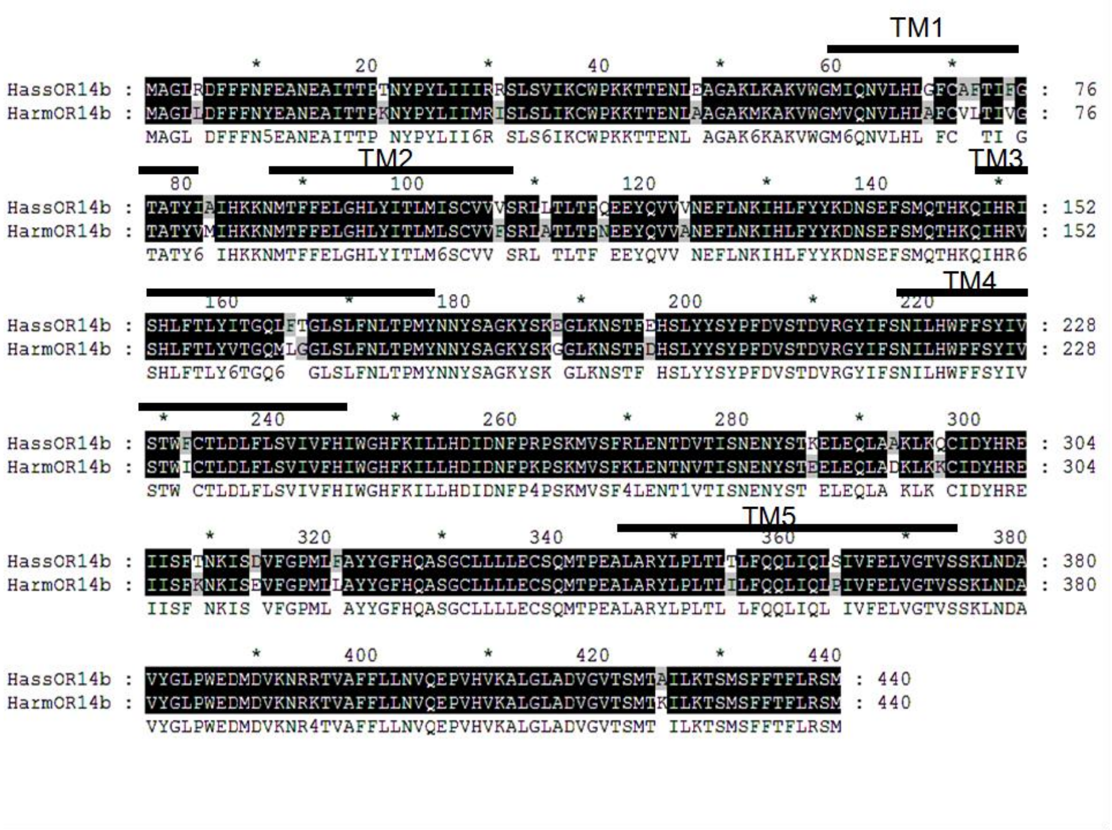

Supplementary Figure 2. Sequence analysis of the homologous candidate pheromone receptors OR14b of *H. armigera* and *H. assulta*

Five putative trans-membrane domains are indicated with black bar and ‘‘TM ‘n’’’ designation, where ‘n’ designates sequential order of the putative transmembrane domain. Trans-membrane domains were predicted by DNAMAN software.
